# A Modified Miniscope System for Simultaneous Electrophysiology and Calcium Imaging *in vivo*

**DOI:** 10.3389/fnint.2021.682019

**Published:** 2021-08-16

**Authors:** Xiaoting Wu, Xiangyu Yang, Lulu Song, Yang Wang, Yamin Li, Yuanyuan Liu, Xiaowei Yang, Yijun Wang, Weihua Pei, Weidong Li

**Affiliations:** ^1^The State Key Laboratory on Integrated Optoelectronics, Institute of Semiconductors, Chinese Academy of Sciences, Beijing, China; ^2^University of Chinese Academy of Sciences, Beijing, China; ^3^Bio-X Institutes, Shanghai Jiao Tong University, Shanghai, China; ^4^CAS Center for Excellence in Brain Science and Intelligence Technology, Shanghai, China

**Keywords:** modified miniscope system, probe, high resolution, electrophysiology recording, calcium imaging

## Abstract

The miniscope system is one of the calcium (Ca^2+^) imaging tools with small size and lightweight and can realize the deep-brain Ca^2+^ imaging not confined to the cerebral cortex. Combining Ca^2+^ imaging and electrophysiology recording has been an efficient method for extracting high temporal-spatial resolution signals in the brain. In this study, a particular electrode probe was developed and assembled on the imaging lens to modify the miniscope system. The electrode probe can be tightly integrated into the lens of the miniscope without increasing the volume, weight, and implantation complexity. *In vivo* tests verified that the proposed modified system has realized the simultaneous recording of Ca^2+^ signals and local field potential (LFP) signal in the hippocampus CA1 region of an adult mouse.

## Introduction

Research in brain function and neural circuit is exceptionally significant for diagnosing, regulating, and treating brain diseases, and it is still a challenge in brain science. The combination of optical imaging and electrophysiology has been developed promptly in the neuroscience field for synchronously observing and recording the brain activities of a specific region.

Early neuroscience research and human clinical studies mainly rely on electrophysiological recordings. This technique allows for the direct recording of neuronal activities (Buzsáki, 2004). The electrophysiology can record the electrical activity of the neuron with a high time resolution (submillisecond) (Fekete and Pongrácz, 2017; Jun et al., 2017) and it is still the gold standard to measure fast neuronal activities (Scanziani and Haeusser, 2009). However, a low spatial resolution limits the electrophysiological recording techniques due to the finite channels of the probe. Besides, single electrophysiology is blind to specific neurons and signal pathways (Park et al., 2011).

The calcium (Ca^2+^) imaging technique provides an option to understand the functional interconnection of a neuron by recording large populations of neurons (Timko et al., 2009; Khodagholy et al., 2011). There are some kinds of devices, such as fluorescence microscope (Kuzum et al., 2014), two-photon microscope (Thunemann et al., 2018; Zatonyi et al., 2020), and fiberscopes (Pochechuev et al., 2018), to realize Ca^2+^ imaging. Nevertheless, the achievable temporal resolution is limited by a slow (millisecond) Ca^2+^-binding kinetics, as the calmodulin-based, genetically encoded fluorescent Ca^2+^ indicators always displayed an extended fluorescence decay time. In other words, Ca^2+^ signal tracking high-frequency action potentials may be limited by Ca^2+^ dynamics (Frostig, 2002; Helassa et al., 2017).

The combination of electrophysiology recording and optical imaging could take advantage of the temporal and spatial resolutions of these techniques, becoming a method for mapping brain activity (Jun et al., 2017). The two-photon microscope imaging technology has the merits of a fast-scanning speed, high-resolution, and three-dimensional local depth imaging and it is widely used in neuroimaging (Zatonyi et al., 2020). Some researchers have developed few kinds of electrode probes to coordinate with this imaging device to obtain electrocorticography (EcoG). For example, Kuzum et al. (2014) utilized transparent graphene microelectrodes based on polyimide and the confocal two-photon microscopy, which realized the electro-optic mapping of slices of the hippocampal tissue. Donahue et al. (2018) demonstrated an extremely flexible (∼4-μm thick) cortical microelectrode array deposited on an optically transparent Parylene-C substrate. The microelectrode array was positioned onto the craniotomy and covered by a coverslip which recognized simultaneous two-photon imaging of the underlying tissue (Donahue et al., 2018). Thunemann et al. (2018) reported a transparent graphene microelectrode technology that eliminated light-induced artifacts. This technology enabled a cross talk-free integration of the two-photon microscopy and optogenetic stimulation cortical recordings in *in vivo* experiments (Thunemann et al., 2018). Zatonyi et al. (2020) presented a novel material composition of a micro ECoG device relying on Parylene HT and indium tin oxide. This device facilitated the two-photon imaging of Ca^2+^ signals and simultaneous recording of the cortical electroencephalogram (Zatonyi et al., 2020). All the aforementioned applications were based on a fixed setup due to the large volume of imaging devices. Therefore, some researchers devoted themselves to develop microscopes of lightweight and miniature volume for studying the behaviors of free-moving animals. For example, miniature two-photon microscopy (Zong et al., 2021), miniature microscopy for single-photon fluorescence imaging (Liberti et al., 2017), and compact head-mounted endoscopy (Jacob et al., 2018) have been developed. The miniscope is also a portable microimaging system consisting of a camera, an optical path system, and a detachable graduated refractive index (GRIN) lens. The detailed construction can be found in the study by Aharoni et al. (2019). The miniscope can easily realize Ca^2+^ imaging in lively free-moving animals. One GRIN lens or multiple lenses of different depths can be implanted simultaneously to observe the connections and interactions between different brain regions. The fluorescent signal can be collected by a camera.

At present, the combined parts of the electrophysiology recording and optical imaging are separated in actuality. The ECoG probe is placed on the cerebral cortex. Then, the mouse is moved under the objective lens of a microscope. It can only image neurons in the superficial layer or isolated tissues of the brain limited by this combination way. Furthermore, the integration of electrophysiology and Ca^2+^ imaging in free-moving animals has not been proposed.

In this study, the lens of the miniscope was transformed from an optical probe to an optical and electrical probe; electrical recording sites were fabricated on the end face of the lens by integrating a special ultrathin transparent probe on the GRIN lens. The electrode probe was tightly integrated into the lens, thus, the modified device could recognize Ca^2+^ imaging and local field potential (LFP) recording in the field of view on the superficial layer of the brain or deep brain by integrated implanted.

## Materials and Methods

### Design and Fabrication of the Electrode Probe

**FIGURE 1:**
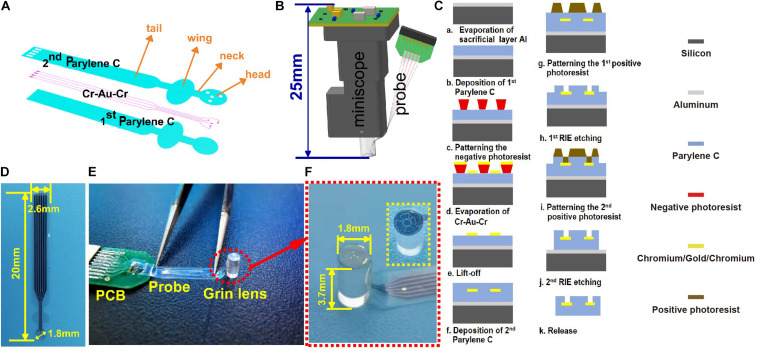
**(A)** The design of the probe; **(B)** the concept diagram of the modified system for simultaneously observing Ca^2+^ and cortical electrophysiology; **(C)** the microfabrication process of the electrode probe from the cross-sectional view; **(D)** the fabricated probe; **(E)** the assembled probe and GRIN lens; and **(F)** the zoom-in view of the imaging and recording regions of the electrode probe as red dotted circles in panel **(E)**. The inset circled by a dotted box is another type of probe designed in the experiment for imaging comparison.

The GRIN lens (H18-S0250-063-NC, GoFoton Nanjing Trading, INC., Nanjing, China) of the miniscope system is a cylinder with a diameter of 1.8 mm and a height of 3.7 mm. The electrode probe profile was designed as shown in Figure 1A to assess the behaviors of the target neurons under the lens. It mainly consisted of the head, neck, wing, and tail. The head was a circle (*Φ* = 1.8 mm). Four circular electrodes of a diameter of 30 μm were distributed on the head around the imaging region to record the LFP. Then, a neck with a width of 800 μm and a length of 800 μm was designed to connect the head and the wing. The wing with a width of 2.6 mm will be glued to the side of the GRIN lens. Thus, the wing length should not be too long to ensure enough remaining height of the lens to insert into the imaging system. All rounded corners in the design were to reduce the force on the stress concentration point. The concept diagram of the integration method is shown in Figure 1B. The head was stuck to the end face of the GRIN lens by a waterproof transparent adhesive. There was a 90° bend at the neck to make the wing stick to the side of the GRIN lens. The rest of the electrode, called the tail, was connected to the printed circuit board for subsequent tests. The total length of the probe was 20 mm.

The electrode probe was fabricated by the microelectromechanical system process, including chemical vapor deposition (CVD), photolithography, lift-off, and reactive-ion etching (RIE). The fabricated device was with a resulting thickness of ∼9 μm. The detailed fabrication process is shown in Figure 1C. (I) A sacrificial aluminum layer with a thickness of 300 nm was evaporated on a single-sided polished silicon wafer. (II) About 4.5-μm Parylene C was deposited (PDS 2010, Specialty Coating System, Inc., Indianapolis, IN, United States) on the silicon substrate as the lower insulation layer. (III) The negative photoresist (AR-N4340, ALLRESIST GmbH, Strausberg Germany) was spin-coated at 4,000 rpm for 20 s and then baked at 110°C for 2 min on the hot plate. Mask aligner (G-33, Chengdu, China) was used for the UV light (365 nm) exposure. The exposure dose of AR-N4340 was 123 mJ/cm^2^. A postexposure bake was conducted at 100°C for 80 s. Then, the solution of the developer (KMP PD238-II, Beijing Kehua Microelectronics Material Co. Ltd., Beijing, China) and deionized water (i.e., 5:2) were used to form the pattern. Cr–Au–Cr with 10–200–10 nm in thickness was evaporated on the wafer. Then, the metal routing pads were formed by immersing the wafer in an acetone bath through liftoff. As an adhesion layer, Cr was used to improve the adhesion of Au and Parylene C. (IV) Then, a 4.5-μm upper insulation layer was deposited by using the CVD method. (V) The positive photoresist AZ-4620 was spin-coated at 4,000 rpm for 20 s on the upper insulation layer as a mask. It was then baked at 90°C for 10 min. The exposure dose of AR-4620 was 490 MmJ/cm^2^. Then, the wafer was immersed in the developer KMP PD238-II for 4 min and post-baked at 110°C for 20 min to form the photolithographic pattern. The pad, the recording sites, and the probe profile were exposed by RIE at 300-W power with O_2_ plasma. (VI) The top layer of Cr was corroded to expose the Au interface. (VII) Then, the probe was released from the silicon wafer by electrolyzing aluminum. The residue that remained on the probe was cleaned by using sulfuric acid. The fabricated probe is shown in Figure 1D. The encapsulated probe is shown in Figure 1E. The modified surface of the lens is shown in Figure 1F. The inset in Figure 1F circled by a dotted box displayed another type of probe (i.e., hollowed probe) designed in the experiment for imaging comparison.

### Transparency Measurements

**FIGURE 2:**
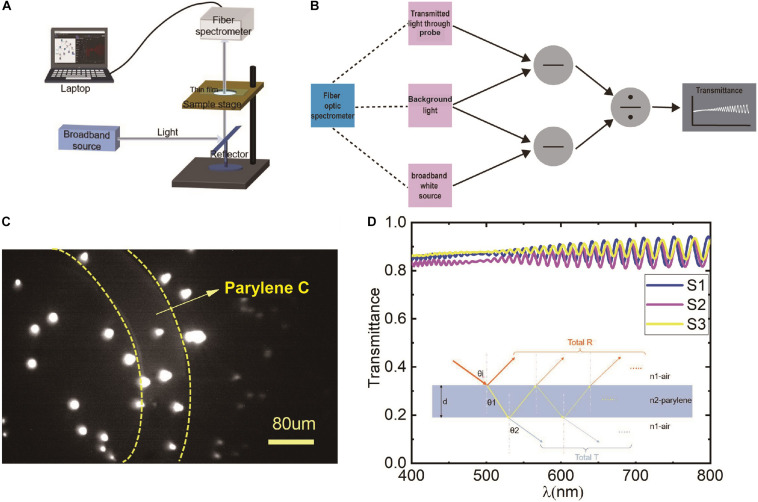
**(A)** The schematic diagram of the transparency measurement; **(B)** the calculation schematic diagram of the light transmittance of the corresponding software; **(C)** fluorescent microspheres imaging results by miniscope taped with a hollowed electrode probe (∼9 μm) *in vitro*, the region between the yellow dotted lines indicates lens taped with Parylene C, the other area indicates imaging without Parylene C; and **(D)** the calculated light transmittance of three samples, S1, S2, S3, respectively. The result of each sample is the average of three transparent areas. The inset indicates the light transmission path at the interface of air and Parylene.

A fiber-optic spectrometer was employed to test the light transmittance of the material in the visible range. The schematic diagram of the test method is shown in Figure 2A. The test system consisted of a broadband white light source, reflector mirror, sample stage, fiber-optic spectrometer, and a computer. First, the intensity of the background light in the test environment was collected and recorded by the fiber-optic spectrometer as *N*_*back*_. Then, the broadband white light source was turned on, and its intensity was collected and recorded by the fiber-optic spectrometer as *S_source_*. Next, the probe was placed between the broadband white light source and the collect fiber to collect the transmitted light that passed through the transparent area of the probe as *S*_*tran*_. Finally, the *N*_*back*_ was removed from *S_source_* and *S*_*tran*_ by subtraction, and the light transmittance of the probe could be calculated by division in the corresponding software by using formula (1); the schematic diagram of the transmittance calculation is shown in Figure 2B.

(1) $$\text{T}=\frac{{\text{S}}_{\mathrm{tran}}-{\text{N}}_{\mathrm{back}}}{{\text{S}}_{\mathrm{source}}-{\text{N}}_{\mathrm{back}}}$$

In this study, three probe samples were measured. Three different areas were tested for each sample. Then, the average light transmittance of each probe was calculated.

### Impedance Measurements

**FIGURE 3:**
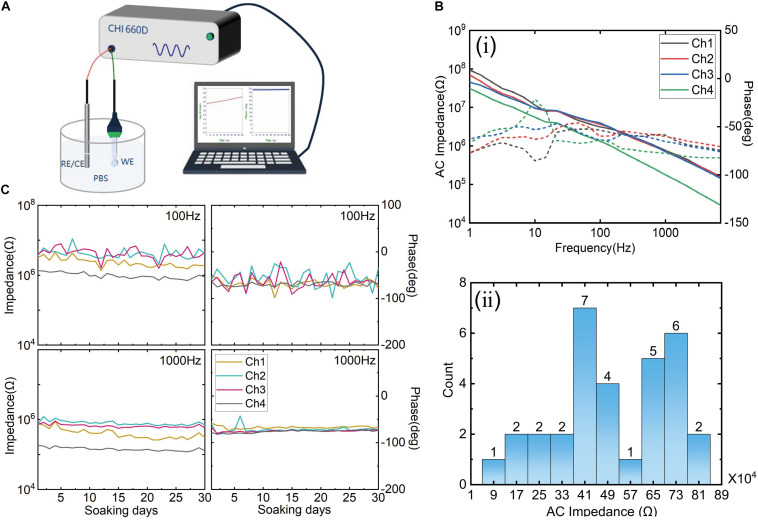
**(A)** The test method schematic diagram of impedance; **(B)** (i) the impedance spectroscopy of one typical sample and (ii) the impedance distribution of eight probe samples with a total of 32 electrodes. **(C)** The aging test results of 30 days; the left column is the impedance fluctuation, and the right column is the phase fluctuation at 100 and 1,000 Hz, respectively.

The electrochemical characterization of the electrode was measured *via* AC Impedance (CHI660D, Chenhua Inc., China). A two-electrode configuration was set up in 0.1-M phosphate-buffered saline (PBS), as shown in Figure 3A. The fabricated electrode acted as a working electrode, and a Pt electrode served as a reference electrode. Sinusoidal AC signals with a voltage amplitude of 5 mV and frequencies from 0.1 to 1,000 Hz were applied to measure the impedance. To evaluate the reproducibility of the probe, 8 probes with a total of 32 electrode impedance (each probe with four electrodes) were tested, and the average impedance and the SD were calculated. Besides, an aging test was performed at room temperature (25 ± 2°C). A typical electrode probe was immersed in PBS solution for 30 days to assess the impedance fluctuation. Impedance was tested every 24 h.

### Electrophysiology and Neuroimaging *in vivo*

According to the guidelines approved by the Committee on Animal Care and Use of Shanghai Jiao Tong University (No. 10773), China, all experiments involving animals were performed. Adult male C57BL/6 mice (aged 8 weeks) were obtained from Beijing Vital River Laboratory Animal Technology Co., Ltd., which were housed singly in a temperature-controlled room with a 12-h light/12-h dark cycle (under light from 7:00 a.m. to 7:00 p.m., 22 ± 2°C) condition.

The implantation protocol here was modified from the previously described protocol (Cai et al., 2016). Briefly, the mouse was anesthetized with isoflurane (4% induction, 1–1.5% maintenance, RWD Life Science) and placed in a custom stereotaxic frame (68055, RWD Life Science, China: Shenzhen, China). Body temperature was maintained with a heating pad. Carprofen (5 mg/kg, ZOETIS, Parsippany, NJ, United States) and dexamethasone (0.2 mg/kg, Jilin Huamu Co., Changchun, Jilin) were administered subdermally during surgery. In addition, 1% Lidocaine (100 μl, Taiyuan Pharmaceutical Co., Taiyuan, China) was applied to the surgical region for pain relief.

**FIGURE 4:**
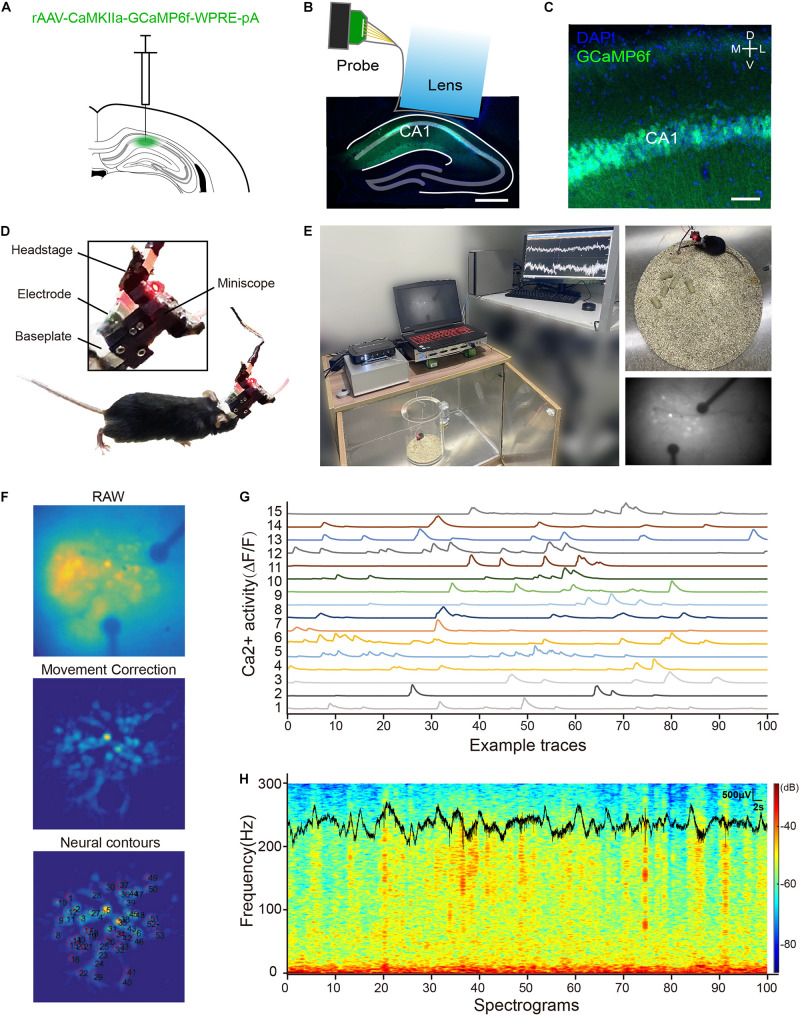
**(A)** A picture of virus injection; **(B)** an image of CA1 region with an assembled electrode probe and GRIN lens implant. Scale bar, 500 μm; **(C)** confocal image of rAAV-CaMKIIa-GCaMP6f-WPRE-pA expression in the CA1 region, GCaMP6f (green), and 4′,6-diamidino-2-phenylindole (DAPI) (blue). Scale bar, 50 μm; **(D)** profile display of electrophysiology and calcium imaging head-fixed interface; **(E)** calcium imaging and local field potential (LFP) recording in mouse CA1 region with an assembled probe and GRIN lens; **(F)** key processing steps of MIN1PIPE-master; **(G)** the traces of spontaneous Ca^2+^ activity from 15 CA1 neurons identified by the MIN1PIPE-master program; and **(H)** the example traces of synchronous LFP and power spectrograms in the CA1 region.

The scalp was disinfected with 75% alcohol and removed to expose the skull. The Bregma and lambda were aligned to set the anterior-posterior plane to a flat level. Then, 500-nl rAAV-CaMKIIa-GCaMP6f-WPRE-pA (PT-0119, BrainVTA, Wuhan, China) were slowly (100 nl/min) injected (100 nl/min, Micro4, WPI, Sarasota, FL, United States) into the dorsal hippocampus CA1 region (AP: –2.1 mm, ML: –2.0 mm, DV: –1.60 mm from skull surface) through a 40-μm glass micropipette attached to microsyringe (7653-01, Hamilton, Nevada; Franklin, MA; Bonaduz, Switzerland). After injection, the needle was left in plac e for 15 min and then slowly withdrawn. Then, the viruses were allowed to infect the neurons and the GCaMP6f expression for 3 weeks. Later, a 2.0-mm diameter circular craniotomy was made using a dental drill up to the site of virus injection. The tissue above the CA1 region was aspirated with a 28-gauge blunt syringe needle linked to a vacuum pump. Artificial cerebrospinal fluid (ACSF) was repeatedly applied to the exposed tissue to prevent drying. The CA1 surface was identified by vertical axon striations and flushed with ACSF until it was clear of blood. The assembled probe and GRIN lens were then slowly lowered to the final depth coordinate (DV: –1.40 mm from skull surface). The diagram is shown in Figure 4B. The lens was fixed with superglue, and then dental cement was applied surrounding the lens with two anchor screws inserted into the skull. The connector of the electrode probe was fixed at the edge of the skull by dental cement. After assembled probe and GRIN lens implantation, a baseplate was mounted on the dental cement cap at the optimal focal distance for imaging. After the surgery, the mouse received carprofen (5 mg/kg) for postoperative analgesia and was allowed to recover for 2 weeks before recording.

After a recovery period, the mouse was placed into a recording chamber. A UCLA miniscope^1^ with a 2.8-g weight was attached to the baseplate and locked with a screw. A super bright blue LED with a 465-nm peak wavelength in miniscope was used as the excitation light source (LXML-PB01-0030, LUMILEDS, San Jose, CA, United States). The radiometric power of the LED is 30 mW. Images were acquired at 30 frames per second and recorded to uncompressed.avi files. Simultaneously, the LFP signals were digitized at 30 kHz by a multichannel recording system (Neurostudio, Jiangsu Brain, Nanjing, China), and then for low pass filtered (i.e., 200 Hz), the continuous signal was used for the LFP analysis. During recording, the miniscope sends TTL pulse signals in 50-ms intervals to Neurostudio as synchronization markers. The data of Ca^2+^ imaging were processed from the raw video files by a custom-written program MIN1PIPE-master (Lu et al., 2018), where videos underwent background subtraction, movement correction, automatic seeds selection, and separation of the region of interest. The LFP data were analyzed using the Neuroexplorer software (Version 5, Nex Technologies, Vienna, Austria).

The mouse was deeply anesthetized and perfused transcardially with PBS (Beyotime, Shanghai, China) and 4% paraformaldehyde (PFA, Sigma, Shanghai, China). The brain was removed, fixed overnight in 4% PFA at 4°C, and dehydrated in 30% sucrose in PBS solution until it was immersed to the bottom. Coronal sections (i.e., 35 μm) were cut using a cryostat (CM3050 S, Leica). The sections were mounted onto the slides with a mounting medium containing 4′,6-diamidino-2-phenylindole (0100-20, SouthernBiotech, Birmingham, AL, United States). The images of GCaMP6f-expressing neurons were obtained using a confocal microscope (TCS SP8, Leica, Buffalo Grove, IL, United States).

## Results

### The Light Transmittance of the Electrode Probe

The average light transmittance of each sample is shown in Figure 2D. The light transmittances were all over 80% in the visible light spectrum from 400 to 800 nm. Besides, we could see the oscillation phenomenon, and the period and amplitude increase gradually with the wavelength. This phenomenon was a result of interference in the thin film (Šantic and Scholz, 2008). A beam of the incident light will produce many different phases of transmitted light, and these transmitted lights interfere with each other to get the total transmission spectrum, as shown in the inset of Figure 2D. The interference formula of the transmitted light is as follows:

(2) 2⁢n⁢d⁢c⁢o⁢s⁢θ⁢i=m⁢λ,m=P⁢o⁢s⁢i⁢t⁢i⁢v⁢e⁢i⁢n⁢t⁢e⁢g⁢e⁢r

In the equation, *n* is the refractive index of the film, *d* is the thickness of the film, *λ* is the incident wavelength of the light, and θ*i* is the refraction angle at the interface between air and Parylene film.

To evaluate the effect of Parylene C on the fluorescent imaging, a hollowed probe with a thickness of ∼9 μm was designed and fabricated simultaneously. The hollowed probe was the same as the abovementioned electrode probe except for the head part. In the head of this probe, a part of the blank area was hollowed. Thus, the imaging could be observed for comparison with/without a probe when the hollowed probe was stuck to the end face of the lens. The hollowed probe is shown in the inset in Figure 1F. Then, the hollowed probe was integrated with the lens and inserted into the miniscope system to obtain the fluorescent imaging *in vitro*. Fluorescent microspheres (e.g., polystyrene microspheres) were used to evaluate the imaging effect. The excitation wavelength of the green fluorescent beads is 488 nm, the emission wavelength is 518 nm, and the particle size is greater than 5 μm. In Figure 2C, the region between the yellow dotted lines indicates the lens with Parylene C. The other area indicates the absence of Parylene C. It can be seen that Parylene C has less impact on imaging.

### The Impedance of the Electrodes

The impedance spectroscopy of a typical sample is shown in (i) of Figure 3B. The average impedance of four channels was about 602 KΩ at 1 kHz, and the phase was around –65°C, indicating a typical gold interface (Ji et al., 2019; Wang et al., 2019). The impedance distribution of eight samples (i.e., 32 electrodes) is shown in (ii) of Figure 3B. The average impedance of 32 electrodes was 501.38 ± 201.15 KΩ at 1 kHz. The impedance range was from 52 to 849 KΩ (at 1 kHz). The range was divided into 10 equal intervals to display the counts of the electrode. For example, the distribution of impedance of 7 electrodes is between 37 and 45 KΩ. The middle of 37 and 45 KΩ was 41 KΩ, marked in the axis of abscissa. The number of electrodes distributing in ranges of different impedance values was counted. It can be seen that only one electrode has an abnormal impedance value (lower than 100 KΩ). This result indicates a good reproducibility of the electrode probe fabrication. Although the impedance of some electrodes was relatively high, it is in an acceptable range. If PEDOT or IrOx modified the interface, the impedance would be decreased, as reported by Musk and Shukla (2019). The aging test results at different frequencies are shown in Figure 3C. The left column is impedance fluctuation. According to the results, it can be seen that the solution-electrode interface had a stable characteristic. The aging test indicated that the fabricated probe could be worked in PBS for at least 1 month. Before *in vivo* experiment, all the electrode probes will be tested. Samples with poor interface characteristics will be discarded.

### Electrophysiology and Neuroimaging *in vivo*

During *in vivo* test, the mouse was injected with the rAAV-CaMKIIa-GCaMP6f-WPRE-pA virus into the dorsal CA1 region of the hippocampus, as shown in Figure 4A. Figures 4B,C show the expression of GCaMP6f in the hippocampus. A miniscope combined with a multichannel recording system was used to image fluorescent Ca^2+^ signals and LFP in the dorsal CA1 region, as shown in Figure 4D. The mouse was housed in a polymethyl methacrylate chamber with a shielded box outside for LFP and Ca^2+^ signals monitoring, as shown in Figure 4E. Figure 4F shows the key steps of Ca^2+^ imaging data processing using MINIPIPE. This result indicates a good imaging effect that the fluorescence change of neurons can be identified and traced. Figure 4G displays the spontaneous Ca^2+^ activity from 15 CA1 neurons by △*F*/*F* in 100 s. The corresponding spectrograms of the synchronous LFP of one electrophysiology recording channel are shown in Figure 4H. These results indicate that the integration of the electrode probe and the lens did not restrict the normal activity of the mouse and the complication of the implantation. Moreover, the real combination of electrophysiology recording and Ca^2+^ imaging was realized.

## Discussion

A novel combination method of electrophysiology recording and Ca^2+^ imaging was proposed in this research. This method could enable the high-spatiotemporal-resolution mapping of the brain in free-moving animals, extending the research scope in neuroscience. The combined system can be implanted in the mouse, which can be free moving, compared with the probe proposed in the studies by Kuzum et al. (2014), Thunemann et al. (2018), and Zatonyi et al. (2020). The probe in the reference has to be placed on a fixed and motionless mouse for imaging. The conductive routing and electrodes in the reference were fully transparent, which did not block the observed view. However, in this study, to simply verify the feasibility of this method, we employed the traditional gold acting as the electrode-tissue interface materials instead of the transparent conductive material (a more complicated fabrication process). The gold electrode would block the observing view, which will hinder the light transmission and may induce artifacts, as shown in Figure 4F. Thus, the gold electrode must be replaced by a transparent conductive material (i.e., graphene/indium tin oxide) in future studies. To study the activities of neurons of a free-moving mouse, a micro-scope was studied, as mentioned in the studies by Liberti et al. (2017), Jacob et al. (2018), and Zong et al. (2021). The imaging in living and moving mouse was realized, but the recording of electrophysiology signals was still a challenge. Traditional large-area ECoG probe was unable to be integrated into the imaging system. Thus, in this study, the probe was designed especially for the imaging system that could be taped to the lens exactly. In conclusion, the main features of this research include (a) a novel integration method different from the traditional combination method; (b) a specially designed and fabricated electrode probe stuck to the GRIN lens of miniscope; (c) an optical probe was transformed to an optical and electrical probe by the special transparent flexible electrode probe; and (d) the integrated implantation was not limited to the surface of the cerebral cortex, which can realize deep brain and multiple brain regions implantation for simultaneous Ca^2+^ imaging and electrophysiology recording.

Additionally, more channels have to be arranged on the head of the electrode probe if the transparent conductive material is employed. The high-density recording is one of the critical aims for the ECoG device to resolve more neuronal activities. Only when the electrode density is higher, can the signal be recorded by multiple channels and synchronized with the change of fluorophores.

In preparation, integrated packaging, and implantation, the electrode probe has the problem of being easy to break. In this study, we used scotch tape for reinforcement. Therefore, it is necessary to choose transparent and stretch-resistant materials in future research in the selection of materials.

Besides, this is preliminary research of the verification of the proposed combination method. Therefore, simply the spontaneous electrophysiology signal and the Ca^2+^ activity were recorded. The correlation between these two signals could not be observed intuitively. Thus, a more detailed and particular *in vivo* experiment with an animal model (e.g., for epilepsy or other stimulus paradigms) should be designed to further verify this modified system.

In summary, the integration method has been proved in the live free-moving mouse for simultaneous Ca^2+^ imaging and electrophysiology recording. We expect that the method presented in this study could be a powerful tool in neuroscience research.

## Data Availability Statement

The raw data supporting the conclusions of this article will be made available by the authors, without undue reservation.

## Ethics Statement

The animal study was reviewed and approved by the Committee on Animal Care and Use of the Shanghai Jiao Tong University, China.

## Author Contributions

XW, XYn, WP, and WL contributed to the conception and design of the study. XW, XYo, YLa, and YLu performed the fabricated process. XYn and LS performed the *in vivo* experiment and *in vivo* data statistical analysis. XW and YWa performed part of the experiments and analyzed the data. XW and WP wrote the first draft of the manuscript. XYn and LS wrote sections of the manuscript. All authors contributed to manuscript revision, read, and approved the submitted version.

## Conflict of Interest

The authors declare that the research was conducted in the absence of any commercial or financial relationships that could be construed as a potential conflict of interest.

## Publisher’s Note

All claims expressed in this article are solely those of the authors and do not necessarily represent those of their affiliated organizations, or those of the publisher, the editors and the reviewers. Any product that may be evaluated in this article, or claim that may be made by its manufacturer, is not guaranteed or endorsed by the publisher.

## Footnotes

1
http://miniscope.org
